# Using U.S. Medicare records to evaluate the indirect health effects on spouses: a case study in Alzheimer’s disease patients

**DOI:** 10.1186/1472-6963-14-291

**Published:** 2014-07-07

**Authors:** Daniel M Gilden, Joanna M Kubisiak, Kristin Kahle-Wrobleski, Daniel E Ball, Lee Bowman

**Affiliations:** 1 JEN Associates, 5 Bigelow Street, Cambridge, MA 02139, USA; 2 Eli Lilly and Company, Lilly Corporate Center, Indianapolis, IN 46285, USA

**Keywords:** Alzheimer’s disease, Dementia, Medicare, Administrative records, Spouses, Medical costs, Indirect costs, Caregivers, Psychological stress

## Abstract

**Background:**

The burden experienced by spouses of patients with Alzheimer’s disease (AD) may have negative consequences for their physical health. We describe here a method for analyzing United States Medicare records to determine the changes in health service use and costs experienced by spouses after their marital partner receives an AD diagnosis.

**Methods:**

We initially identified all beneficiaries in the 2001–2005 Medicare 5% sample who had multiple claims listing the ICD-9 diagnostic code for AD, 331.0. The 5% sample includes spouses who share a Medicare account with their marital partners because they lack a sufficient work history for full eligibility on their own. A matched cohort study assessed incremental health costs in the spouses of AD patients versus a control group of spouses of non-AD patients. Longitudinal and cross-sectional analyses tracked the impact of a patient’s AD diagnosis on his or her spouse’s healthcare costs.

**Results:**

Our method located 54,593 AD patients of whom 11.5% had spouses identifiable via a shared Medicare account. AD diagnosis in one member of a couple was associated with significantly higher monthly Medicare payments for the other member’s healthcare. The spouses’ elevated costs commenced 2 to 3 months before their partners’ AD diagnosis and persisted over the follow-up period. After 31 months, the cumulative additional Medicare reimbursements totaled a mean $4,600 in the spouses of AD patients. This excess was significant even after accounting for differences in baseline health status between the cohorts.

**Conclusion:**

The study methodology provides a framework for comprehensively evaluating medical costs of both chronically ill patients and their spouses. This method also provides monthly data, which makes possible a longitudinal evaluation of the cost effects of specific health events. The observed correlations provide a coherent demonstration of the interdependence between AD patients’ and spouses’ health. Future research should examine caregiving burden and other possible factors contributing to the AD spouses’ health outcomes. It should also extend the method presented here to evaluations of other chronic diseases of the elderly.

## Background

Cost-of-disease studies in dementia frequently group together the various dementia types because of difficulty in distinguishing between different diagnoses in the absence of detailed clinical assessments. Nonetheless, it is important to understand the full costs of Alzheimer’s disease (AD) since it is the most common dementia and its prevalence is increasing rapidly [1]. AD furthermore has its own particular disease profile [2,3]. It is associated with specific care interventions (direct costs) [4,5] and makes particular demands on caregivers (indirect costs) [6,7]. The study of AD’s secondary effects has proved especially problematic: Past attempts to calculate incremental health costs among informal dementia or AD caregivers have varied widely because study inclusion criteria, control groups and sources of cost data are not consistently constructed [8,9].

Spouses of AD patients experience both a large caregiving burden and special psychosocial issues [10,11]. A study of caregiving wives of patients with either AD or vascular dementia reported that their caregiving time averaged 8 hours per day after 3 years [12]. In addition, spouses’ intimate daily connection to their AD partners triggers a growing sense of grief as their partners’ ability to interact declines [13,14]. Social isolation and financial difficulties also commonly ensue [15,16].

The best approach to quantifying these stressors’ impacts on caregivers and particularly spousal caregivers remains unresolved. A large number of studies report that the stress experienced by caregivers results in health declines, which especially affect elderly caregivers of dementia patients [17-19]. However, few studies consider the spousal role in its entirety, and those that do still confound AD with dementia in general [20,21]. These studies also were subject to limitations in matching the spousal and control populations that may have masked healthcare cost differences. A recent study of healthcare costs matched household members of AD patients with a control population but did not separately identify spouses [22].

To help address gaps in health economic knowledge on the spousal experience, we propose a Medicare claims-based method for looking at AD patients, their spouses and the joint impact the two have on direct medical spending for elderly adults by the United States’ Medicare fee-for-service program. Medicare claims present the possibility of identifying a large number of spouses of AD patients and finding a closely matched control group since detailed diagnosis, service utilization and cost data are available for each group. Because of the large number of detailed claims records accumulated over many years, it is possible to conduct a longitudinal investigation to assess the impact of AD diagnosis on spouses’ health. As described here, this claims-based approach provides a model for studying the effect of other chronic conditions on spousal healthcare service utilization and costs.

## Methods

### Identifying persons with Alzheimer’s disease

Advancing age is a strong predictor of AD onset, with the prevalence of diagnosed AD increasing exponentially after the age of 65, from approximately 3% of persons aged 65–74 to just over 30% of persons aged 85+ [23]. U.S. Medicare claims records therefore are an appropriately representative source for estimating the impact of AD patients and their spouses on the US healthcare system.

The U.S. Centers for Medicare and Medicaid Services (CMS) makes available the claims records for a random 5% sample of Medicare beneficiaries for research purposes [24]. This 5% sample encompasses approximately 2 million people representative of the entire US elderly and permanently disabled population. Not counting deaths, 99.5% of the population is carried over during CMS’s annual updates.

We identified our AD population by evaluating all beneficiaries in the 2001–2005 Medicare 5% sample who had claims listing a primary diagnosis of AD (ICD-9 diagnosis code 331.0). Two corroborating AD diagnoses appearing as the primary diagnosis in outpatient claims or one corroborating AD diagnosis listed in a hospitalization claim were required to ensure the robustness of diagnosis. These corroborating healthcare encounters had to occur in the two years following the month of the initial diagnosis (which defined the index date). Other dementia-related codes were not taken into consideration so as to achieve greater diagnostic specificity for AD within the cohort.

Detailed Medicare claims records are available only for the months in which beneficiaries are enrolled in the traditional Medicare fee-for-service (FFS) coverage [25]. This study therefore excluded AD patients and their spouses if they had Medicare managed care coverage during their index month or for ≥6 months in the pre-index year.

### Identifying spouses

Once we had constructed a corroborated AD population, we proceeded to assign spousal relationships. We used a method similar to the one described by Iwashyna et al. [26,27]. It takes advantage of a special feature of the Medicare beneficiary numbering system to accurately identify married couples.

CMS assigns each new Medicare beneficiary an 11-digit identification code known as the Medicare Health Insurance Claim Number, or HICN. The first nine digits are the Claim Account Number (CAN), which is the same as the Social Security Number if the beneficiary is entitled to Medicare based on his or her own work history. Persons without sufficient work history for full eligibility (usually ten years) may receive Medicare benefits through their spouses. These individuals are assigned the same CAN as their spouse, but the last one or two characters of their HICN are different. These characters represent the Beneficiary Identification Code (BIC). Special BICs identify spouses who share an account established through their partner’s work history. CMS selects the Medicare 5% sample based on the CAN, effectively guaranteeing that primary beneficiaries and dependent spouses are selected together.

Using this approach excludes couples in which both members are eligible for Medicare due to their separate work histories. In those situations, each member has a different CAN, and chance co-selection in the 5% Medicare sample would be undetectable without further information.

### Cross-sectional and longitudinal matched control cohorts

After identification of the AD spouse study population, the Medicare records further allowed us to match the AD spouses to a control cohort married to partners without AD. Our selection and matching approach yielded four groups created sequentially: AD patients, AD spouses, matched control spouses and non-AD spouses of matched controls. The comparisons of interest described here are between the AD spouses and control spouses only.

The marriage identification and inclusion criteria were the same for the controls as for the AD spouses except that the controls’ marital partners could not have any record of an AD diagnosis. Each control and AD spouse was then matched according to race/ethnicity, age category and urban/rural residency status. Matching by comorbidity was not done because it would interfere with the study’s examination of the correlation between AD spousal status, certain related diagnoses and their impact on overall costs. The controls received the same index date as their matching AD spouses (i.e., the AD patient’s initial diagnostic date).

We compared the AD spouse and control cohorts cross-sectionally and longitudinally. For the cross-sectional analyses, we constructed in each study year (2001, 2002, 2003, 2004 and 2005) a matched comparison cohort for all identified AD spouses. To be included in the annual spousal profiles, members of the AD and comparison couples had to have at least six months of Medicare FFS records within the given year.

In order to support a longitudinal analysis, matching controls also were selected for the spouses of newly diagnosed AD patients first identified in each study year. AD patients were required to have at least the 12 previous months of Medicare FFS claims devoid of AD diagnoses to qualify for each year’s incident AD population, a criterion that did not apply to the other study defined populations. For the AD spouse-control matched pairs, the observation period commenced six months before the index date and continued until both members of the pair had died or lost FFS eligibility.

It was not possible to observe a prior diagnostic “clean period” for any of the AD patients identified in the first study year, i.e. 2001. These patients were assigned to the cross-sectional analysis only.

### JEN frailty index

We found it necessary to include a covariate indicating each person’s pre-index well-being when constructing regression models of the AD spouse and control cohorts’ healthcare costs. Finding an appropriate marker for patients’ overall health and physical status poses difficulties because of Medicare claims records’ lack of clinical data. In addition, the scope of potential comorbidities in this elderly study population is not adequately represented in a standard comorbidity index such as Charlson or its adaptations [28,29] that cover only a limited number of hospital diagnoses. Another option, stratifying by specific comorbidities, would have been quite challenging statistically. We chose instead to employ the broad-ranging JEN frailty index (JFI). The JFI takes into account claims filed by all providers, not just hospitals. It is therefore particularly useful in patients with complicated clinical profiles. Furthermore, this index was developed for specific use in Medicare/Medicaid database analyses as part of the Medicare/Medicaid Integration Project, funded by the Robert Wood Johnson Foundation [30]. We have found it to be significantly correlated with Medicare and Medicaid expenditures as well as mortality [31,32].

The almost 1,800 diagnoses included in the JFI are classified into 13 condition categories. The 13 condition categories are designed to provide an indication of individual patient impairment. They include minor ambulatory limitations, severe ambulatory limitations, cognitive developmental disability, chronic mental illness, dementia, sensory disorders, self-care impairment, syncope, cancer, chronic medical disease, pneumonia, renal disorders and systemic disorders (e.g. septicemia). A patient’s personal index is derived by counting the presence of these condition categories in the previous year’s diagnostic claims.

### Statistical analyses

The focus of this report is to describe the utility of Medicare claims records for identifying spouses and studying the secondary health effects they experience when their partners have chronic disabling illness, AD in particular. To indicate the effectiveness of this approach, we discuss here the descriptive healthcare utilization and cost comparisons that we obtained. We also mention the primary result of a multiple regression analysis of healthcare costs and utilization. This analysis, constructed in stepwise fashion, included as covariates socioeconomic status, national region of residence, index year, select pre-index (but not post-index) chronic conditions, annual JFI level and status as AD spouse or control. These more extensive multivariate cost and illness analyses are the subject of a conference presentation [33] and a manuscript in preparation.

### Ethical approval

The study was conducted in accordance with a data use agreement from the U.S. Centers for Medicare and Medicaid Services. The study received a waiver of review from the New England Institutional Review Board on the basis that the study’s necessary use of protected health information (PHI) presented no more than minimal risk to individuals’ privacy.

## Results

### Characteristics of the study population

The annual profiles of the entire corroborated AD population are shown in Table 1. We found a total of 54,593 patients over the 5 study years with corroborated AD. The annual AD population averaged 32,293. Mean age was of 82.1 years.

**Table 1:** Annual profiles of corroborated AD patients, 2001-2005

**Population annual characteristics**	**2001**	**2002**	**2003**	**2004**	**2005**
**Number**	23,038	30,935	35,392	37,500	34,601
Observed AD prevalence^1^	1.6%	2.1%	2.3%	2.4%	2.2%
**Gender**					
Male	28.9%	28.9%	29.3%	29.5%	29.2%
Female	71.2%	71.1%	70.7%	70.5%	70.8%
**Age in year** (<1% in below 55 ages)					
Age 55-64	1.1%	1.1%	1.2%	1.2%	1.2%
Age 65-74	12.4%	11.6%	11.0%	10.7%	10.0%
Age 75+	86.3%	87.0%	87.5%	87.8%	88.5%
Mean age	82.41	82.72	82.91	83.03	83.36
**Race/ethnicity**					
White	87.5%	87.0%	86.7%	86.5%	86.3%
Black	9.0%	9.3%	9.4%	9.5%	9.5%
Other race/ethnicity	3.5%	3.7%	3.9%	4.1%	4.2%
**Geographical region**					
Northeast	20.2%	20.2%	19.9%	19.8%	19.6%
Midwest	26.0%	25.2%	25.1%	24.8%	24.8%
South	40.0%	40.6%	40.7%	41.0%	41.0%
West	12.0%	12.2%	12.4%	12.7%	12.9%
Other	1.9%	1.8%	1.8%	1.8%	1.7%
Urban	72.5%	72.8%	73.1%	73.4%	73.5%
Rural	24.8%	24.5%	24.1%	23.7%	23.6%
Unknown	2.7%	2.7%	2.8%	2.9%	2.9%
**Patient status**					
Nursing home at index^2^	39.5%	31.8%	26.6%	22.5%	20.3%
Nursing home entry during follow-up^2^	11.2%	13.4%	12.1%	11.3%	10.9%
Death during follow-up	68.3%	60.2%	48.4%	34.6%	20.3%
Mean follow-up months	34.6	35.6	35.7	33.9	32.4
**Identified spouses**					
Number	2,581	3,234	3,606	3,717	3,184
Percent of total AD patients	11.2%	10.5%	10.2%	9.9%	9.2%
**Incident AD diagnoses**^**3**^					
Number	N/A^4^	9,447	8,728	7,758	3,518
Percent of total AD patients	N/A^4^	30.5%	24.7%	20.7%	10.2%
**Identified incident AD spouses**					
Number	N/A^4^	1,077	975	836	342
Percent of total AD patients	N/A^4^	3.50%	2.80%	2.20%	1.00%

^1^Percentage of listed year’s Medicare fee-for-service population.

^2^To be considered a nursing home resident, a patient’s Medicare Part B claims had to record a pattern of nursing home-specific evaluation and management codes and/or nursing home place of service. This pattern had to be indicative of long-term nursing home residency rather than typical post-acute care.

^3^Incident cases have ≥12 months of Medicare fee-for-service eligibility prior to their first AD diagnosis.

^4^Lack of data before 2001 meant that there could be no determination of incidence during that year.

Out of the total AD population meeting our selection criteria, 6,291 (11.5%) had identifiable spouses (i.e. those without appreciable independent work history). After excluding couples in which one or both members were members of Medicare managed care plans for ≥6 months in the pre-index year, the AD spousal population was reduced by 11%. We referred to the 2000 US census marital status data [34] and the age and sex breakdown of our eligible AD population to establish the estimated number of our AD population that would be expected to be married. On the basis of the census data, the shared CANs are estimated to have flagged 27% of the Medicare 5% sample’s eligible married couples with one AD member (Table 2).

**Table 2:** Spousal identification rates for the corroborated Alzheimer’s disease population and spousal identification rates, 2001-2005

**Sex**	**Total corroborated AD patients**^**1**^	**Number of study-identified spouses**^**1**^	**Expected number of spouses**^**2**^	**Study’s rate of spousal identification**
Male	16,788	3,188	11,602	27.5%
Female	37,805	3,103	11,848	26.2%
Study total estimate	54,593	6,291	23,450	26.8%

^1^From the Medicare 5% sample, with both members of the spousal dyad enrolled in the traditional Medicare fee-for-service plan during the index month.

^2^Weighted by age according to the 2000 US Census marital status data.

SOURCE: Medicare 5% Sample, 2001–2005.

The initial 2001 cohort (n = 23,038) also was substantially lower than in the other years (Table 1) because we had no ability to look retrospectively at the records and identify patients receiving care related to an AD diagnosis before but not during 2001. It is likely that many of these AD-diagnosed Medicare beneficiaries reappeared in the 2002 AD cohort. From 2002 on, such patients received enough repeated care to meet our corroborated AD criteria. The lack of follow-up time past 2005 limited our ability to corroborate AD patients after their initial diagnosis. This restriction reduced the incident AD population observed in 2005.

Table 3 compares the baseline demographics of the identified AD spouses and their matched controls. As intended, the AD spouses and their matched controls had no significant differences in demographics.

**Table 3:** **Pooled annual cohorts: baseline demographics, 2001-2005**^**1**^

	**AD spouses (N = 6,291)**	**Matched controls (N = 16,322)**^**2**^
**Gender**		
Male	49.3%	49.3%
Female	50.7%	50.7%
**Race/ethnicity**		
White	90.0%	90.0%
Black	5.5%	5.5%
Hispanic	3.0%	3.0%
Other race/ethnicity	1.5%	1.5%
**Ages**		
Age 60-64	0.1%	0.1%
Age 65-69	5.3%	5.3%
Age 70-74	14.7%	14.7%
Age 75-79	25.8%	25.8%
Age 80-84	29.4%	29.4%
Age 85+	24.8%	24.8%

^1^The AD spouse and control cohorts were matched on the basis of gender, age category, race/ethnicity, and urban status. Represented here is the entire pooled study population – incident, pre-existing and unknown AD cases with their spouses and the control population with their marital partners.

^2^The total population from 2001 to 2005. When creating the cross-sectional annual cohort profiles, AD spouses were rematched each year to new controls.

### AD spouses’ health care costs

A sizable cost difference occurred between the cross-sectional AD spouses and matched control cohorts (Table 4). Mean annual Medicare expenditures in the two cohorts were $8,206 (95% CI: $7,967, $8,445) and $6,640 (95% CI: $6,414, $6,866), respectively (*P* < 0.001). The mean total Medicare expenditures are large relative to the medians in both populations, indicating that high-cost outliers – whose records include substantial hospitalization – considerably affect these means.

**Table 4:** **AD spouses vs. matched controls: Medicare expenditures, 2001-2005**^**1**^

	**AD spouses**	**Matched controls**	***P*-value**
**Pooled annual cohorts (N = 6,291)**			
**Observation time**			
Median Medicare FFS months/year	12	12	
Mean Medicare FFS months/yr. (95% CI)	11.83 (11.82, 11.84)	11.85 (11.83, 11.86)	NS
**Medicare expenditures**			
Median total expenditures/year	$2,197	$1,658	
Mean total expenditures/yr. (95% CI)	$8,206 ($7,967, $8,445)	$6,640 ($6,414, $6,866)	<.001
Mean total PMPM	$694	$561	
**Incident cohorts (N = 2,987)**			
**Multiple regression analysis**^**2**^			
Impact on Medicare expenditures (95% CI), AD spouse vs. control	29% (18%, 42%)		<.001

^1^Spouses and controls were matched by gender, age category, race/ethnicity and urban/rural county status. The cohorts were restricted to spouses who, with their partners, were Medicare fee-for-service beneficiaries for at least 6 months of the follow-up year. The profiles for each year’s AD spouse and control populations were merged with the profiles for the corresponding cohorts for the other years in the observation period. The annual profiles were calendar-year specific and not linked to an AD diagnosis date.

^2^Result of a log-transformed regression model of Medicare expenditures that adjusted for socioeconomic status, US region of residence, hospitalization during the index month, JFI level during the index month, select pre-index chronic conditions, and length of follow-up. This analysis was restricted to couples in which both the AD patient and spouse were community-dwelling during the observed index month. Follow-up does not include the index month (all patients were required to survive the index month in this analysis).

FFS = fee-for-service; SD = standard deviation; JFI = JEN Frailty Index; PMPM = per Medicare beneficiary per month; CI = confidence interval.

**Figure 1:**
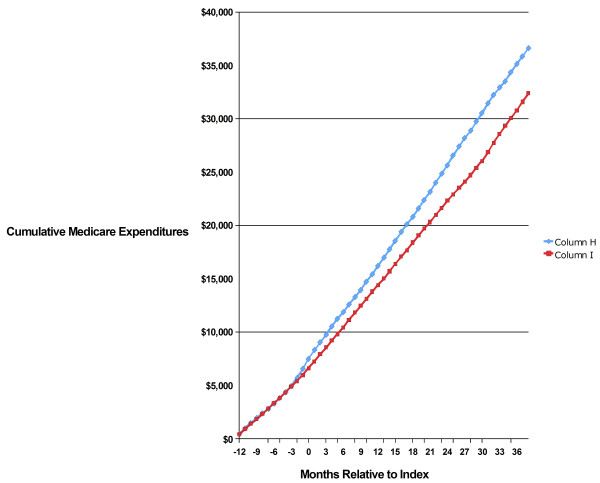
Mean Cumulative Medicare Expenditures, Incident AD Spouses vs. Controls, 2002–2005.

In the longitudinal cohorts, a regression analysis (Table 4) adjusted for pre-index factors that drive healthcare costs to high levels. Even with these adjustments, AD spousal status was associated with an estimated 29% independent cost impact on Medicare expenditures (*P* < 0.001).The matched spousal longitudinal cohort comparison made it possible to observe both the overall cost outcome and the timing of Medicare expenditures relative to the index date. We found that the AD spouses and the controls incur virtually identical Medicare expenditures until 2 to 3 months prior to their partners’ AD diagnoses (Figure 1). The difference in mean cumulative Medicare expenditures increased from this point onward until it reached $4,600 at 31 months after the index date. Expenditures beyond month 31 were similar overall. In our 2002–2005 observation period, only about a third of the incident study population had ≥36 months of data available (all of them incident in 2002). Longer follow-up with more patients is necessary to understand the trajectory of costs beyond 30 months post-index.

## Discussion

The methodology described in this report presents a novel approach to better quantifying the burden on the healthcare system represented by the spouses of patients with AD or other debilitating chronic conditions. By linking spouses with shared CANs in the Medicare 5% sample and creating a control spouse cohort, the impact of an AD diagnosis on the spousal dyad was explored cross-sectionally and longitudinally. The temporal association between the patients’ AD diagnosis and the increase in Medicare expenditures for their spouses as compared to controls illustrates the strength of our method. That relationship underscores the impact of the AD diagnosis on spouses’ health, independent of a couple’s joint lifestyle or other common factors that might contribute to the AD spouses’ medical costs. The ability to track the details of cost and service utilization on a monthly basis is yet another advantage to utilizing Medicare records rather than relying on self-reports of healthcare utilization. Despite such benefits, several complex issues concerning the validity of our approach deserve further examination.

### AD ascertainment through Medicare claims

Medicare claims records are robust since they list all types of services, including those performed on both an inpatient and outpatient basis. Moreover, the need to receive payment for their services disciplines providers to be complete when filing these claims.

Concern nonetheless remains about the reliability of using Medicare claims to summarize the impact of AD and other dementias on resource utilization. Even when an AD-related diagnosis is appropriate, providers faced considerable hurdles obtaining Medicare payment during our study period [35-37]. The literature suggests a physician and hospital bias toward filing claims based on more clear-cut and easily reimbursable diagnostic codes related to patients’ physical comorbidities rather than on patients’ cognitive impairments [35,37].

Two studies have examined Medicare data contemporaneous with our study period [38,39]. One study included patients with presenile/senile dementia diagnostic codes in their claims as well as those with 331.0 for AD [39]. It used only survey responses to validate these diagnoses. The other study [38] carried out formal clinical assessments to determine who had AD, other forms of dementia or mild cognitive impairment in the Aging Demographics and Memory Study (ADAMS) cohort of 856 older Americans.

After reviewing the cohort’s Medicare claims, the ADAMS investigators concluded that the sensitivity and specificity of those claims for capturing an AD diagnosis was respectively, 64% and 95%. By comparison, Medicare claims sensitivity and specificity for all-cause dementia was 85% and 89%. Identifying AD patients through Medicare claims is thus a very selective strategy but misses a substantial percentage of patients, probably those with atypical or mild cases.

The present study’s AD population represents a core of easily identified cases receiving recurring AD-related care. Broadening our AD definition could have increased the study population by as much as two-fold [40,41], but including less severe and more ambiguous cases of AD would have weakened our ability to investigate AD-specific spillover effects on spousal health. Comparing the relationship between various coding patterns and clinical signs of cognitive impairment would help in the development of more accurate algorithms for parsing Medicare claims. That examination is outside our current scope.

### Characteristics of the identified spouses

Since the goal of this study was to evaluate the secondary effects of AD on spouses, our ability to identify only a quarter of the potential spouses becomes important. In making this identification, we took advantage of a wrinkle in the Medicare beneficiary numbering system that identifies spouses without their own retirement accounts through their partners’ Social Security numbers. Medicare automatically includes both such spouses when constructing its 5% beneficiary sample.

Our method identified many primary Medicare beneficiaries who were the spouses (primarily husbands) of dependent partners diagnosed with AD. It omitted households in which both spouses had large enough prior earnings to qualify for Medicare on their own. Women with substantial work histories make up a growing proportion of Medicare beneficiaries, however, and study of spousal effects in two wage-earner couples is becoming more important.

It is possible that spouses with substantial work experience react more resiliently to their partner’s AD. They may have greater financial resources and feel more self-empowered than a spouse with little outside work experience. Conversely, a spouse who has spent a majority of time at home may be more familiar with and/or less burdened by home-based caregiving. Our study could not be sensitive to these possibilities.

An additional 25% of elderly Medicare spouses could be captured when they apply for widow benefits and emerge as married beneficiaries in the Medicare denominator (demographic) data files [26]. Employing this method for spousal healthcare cost and utilization studies would require claim records for the entire Medicare elderly population during the study years plus demographic data for many years beyond. These files were not available to us.

A still more complete spousal identification would involve linking AD case information with address records for each year and matching cohabitants via their common addresses. This method would create a complete file that includes all cohabiting partners, whether they are married or not, while excluding married couples who are separated (a small proportion of elderly spouses [34]). We could then describe in depth the differences between spouses according to their previous work history.

Distributing Medicare address files raises privacy concerns, however, and CMS usually does not release this information. Even if the agency did allow access, the address records would present a significant processing challenge because they lack a standard format. Another challenge would be excluding elderly cohabitants who are simply relatives or roommates rather than spouses.

### Spouses first or caregivers?

Identifying the entire spousal population solely through Medicare claims would still yield no practical way to determine the extent of their caregiving role. A considerable literature on AD and other disabilities does document that cohabiting spouses are usually the chief caregiving resource when they are not incapacitated themselves [12,42-45]. National U.S. surveys have found that up to 80% of spouses living with elderly disabled partners devote substantial time to caregiving tasks [44,45].

This overall spousal caregiving burden does not provide the basis for individual risk analysis, however, and even knowing the caregiving obligations of each spouse would be insufficient to predict their health risk. The nature of a couple’s relationship influences whether caregiving is experienced as fulfilling or a strain, and that subjective interpretation in turn can affect health and mortality [46-48]. The nature of the spousal relationship also independently affects spouses’ mortality when their partners become seriously ill or die [11,49]. These observations are conspicuously true of cognitive impairment: Dementia in one spouse greatly increases the risk of dementia in the other, and spousal closeness decreases dementia progression in the affected partner [50,51].

Spouses’ interrelated health outcomes may be influenced by a couple’s shared environment as well as to their emotional ties. It will not be possible to capture the individual, granular details of this phenomenon through claims records alone. Our method nonetheless allows researchers to show the population-level patterns that mirror the underlying individual interactions.

The question also arises as to whether spouses of patients with other chronic diseases would experience similar increases in healthcare costs. There are rare examples of this phenomenon existing for other disease states [52] or in association with widowhood from decedents of other chronic conditions [10]. The goal of our study was to find a connection between spouses’ healthcare status and their partners’ AD diagnosis while statistically controlling for a broad spectrum of impairments with the JFI. This temporal association is illustrated in Figure 1. One can speculate that AD’s particular behavioral and cognitive effects are especially stressful for spouses, both emotionally and in terms of caregiving burden. It will be a fruitful line of research to compare the spousal results presented in Table 4 and Figure 1 with results obtained for other chronic diseases. The methods we establish here can form the basis for such research.

### Comprehensiveness of costs

Medicare FFS data includes claims related to outpatient and inpatient services, short-term skilled nursing home stays, hospice, select home health services, physician/medical professional services, and durable medical equipment. It does not include certain behavioral health services, many home-care services, or long-term nursing home residencies. Other omissions are payments by private supplementary insurance, Medicaid or pre-2006 outpatient pharmacy costs. So long as the study focuses on comparing relative costs in carefully defined exposed and unexposed cohorts, restricting the quantitative results to Medicare FFS expenditures will not make a qualitative difference in its conclusions. Analyses of data linked from the different sources described above would further advance the research reported here.

## Conclusions

Examining the claims records of married Medicare beneficiaries is a valid, effective approach for studying the interrelationship between spousal healthcare costs and a partner’s AD diagnosis. We conclude that the Medicare claims records provide detailed findings specific for a core group of patients with long-term AD and that these records have the extra advantage of supporting analyses over time.

Our data is less specific for the association between AD caregiving and adverse health effects in the spouse. We have no information on the degree of caregiving provided by each spouse or other informal or professional aides.

Additional research on the characteristics of spousal caregiving would better support our methodology, as would a system that allowed identification of a broader range of Medicare spousal beneficiaries. Nevertheless, spousal studies are possible with the available data. The observed emergence of elevated health service costs among spouses just before their partners’ AD diagnoses is a strong indicator that our methods for identification and matching have a rational basis.

The results constitute a thorough, coherent narrative on the interdependence of spouses’ and AD patients’ health. They show that reciprocal health effects are substantial and should be included in healthcare planning as the population becomes older and AD and other age-related conditions increase in prevalence. The study methodology additionally provides a framework for assessing the value of indirect as well as direct benefits of effective treatments. Further work will determine how useful this methodology is for other chronic, incapacitating diseases of the elderly.

## Abbreviations

AD: Alzheimer’s disease; ADAMS: Aging demographics and memory study; BIC: Beneficiary identification code; CAN: Claim account number; CI: Confidence interval; CMS: Centers for Medicare and Medicaid services; FFS: Fee-for service; HICN: Health insurance claim number; ICD-9: International classification of diseases, ninth revision; JFI: JEN frailty index; PHI: Protected health information; PMPM: Per Medicare beneficiary per month; SD: Standard deviation.

## Competing interests

DMG and JMK were consultants on this study and previous ones for Eli Lilly and Company, which is developing new treatments for Alzheimer’s disease. KKW, DEB and LB are employees of Eli Lilly and Company and also hold stock in the company. The authors have no other financial or non-financial competing interests.

## Authors’ contributions

DMG and JMK conceived this study and its methodological approach. JMK also adapted the data and was responsible for the statistical analysis. KKW, DEB and LB contributed to the design of the study and the critical interpretation of the analysis. All authors participated in drafting and revising the study. Each read and approved the final manuscript.

## Pre-publication history

The pre-publication history for this paper can be accessed here:

http://www.biomedcentral.com/1472-6963/14/291/prepub
